# Measurement Matrix Optimization for Compressed Sensing System with Constructed Dictionary via Takenaka–Malmquist Functions

**DOI:** 10.3390/s21041229

**Published:** 2021-02-09

**Authors:** Qiangrong Xu, Zhichao Sheng, Yong Fang, Liming Zhang

**Affiliations:** 1 Key Laboratory of Specialty Fiber Optics and Optical Access Networks, Joint International Research Laboratory of Specialty Fiber Optics and Advanced Communication, Shanghai Institute for Advanced Communication and Data Science, Shanghai University, Shanghai 200444, China; hahah@shu.edu.cn (Q.X.); zcsheng@shu.edu.cn (Z.S.); 2 Department of Computer and Information Science, Faculty of Science and Technology, University of Macau, Macau 999078, China; lmzhang@um.edu.mo

**Keywords:** compressed sensing (CS), sparse representation, the Takenaka–Malmquist (TM) functions, mutual coherence, equiangular tight frame (ETF)

## Abstract

Compressed sensing (CS) has been proposed to improve the efficiency of signal processing by simultaneously sampling and compressing the signal of interest under the assumption that the signal is sparse in a certain domain. This paper aims to improve the CS system performance by constructing a novel sparsifying dictionary and optimizing the measurement matrix. Owing to the adaptability and robustness of the Takenaka–Malmquist (TM) functions in system identification, the use of it as the basis function of a sparsifying dictionary makes the represented signal exhibit a sparser structure than the existing sparsifying dictionaries. To reduce the mutual coherence between the dictionary and the measurement matrix, an equiangular tight frame (ETF) based iterative minimization algorithm is proposed. In our approach, we modify the singular values without changing the properties of the corresponding Gram matrix of the sensing matrix to enhance the independence between the column vectors of the Gram matrix. Simulation results demonstrate the promising performance of the proposed algorithm as well as the superiority of the CS system, designed with the constructed sparsifying dictionary and the optimized measurement matrix, over existing ones in terms of signal recovery accuracy.

## 1. Introduction

Sparse representation has become a powerful tool, as it can efficiently model signals to facilitate compressing and processing [1,2,3]. Due to the redundancy in the majority of signals in nature, transforming the original signal to a sparse or compressed version through a certain domain is meaningful for reducing costs in transportation and storage. In this case, the signal can be expressed as a linear combination of a few given basis functions, which indicates that most of the representation’s coefficients are zero or close to zero.

Compressed sensing (CS), as one of the most attractive emerging fields in recent years, has shown promising results in many applications, such as channel or spectrum estimation [4,5], cognitive radio communications [6], color pattern detection [7,8], and people-centric sensing [9]. CS states that signals which have sparse structures on an appropriate domain can be effectively recovered from the underdetermined linear projections. However, it is often the case that signals of interest are not exactly sparse but compressible. Projecting the signal into its proper sparse domain to obtain a sparser structure is essential in CS [10]. Hence, one critical issue in sparse representation is how to choose the dictionary (as well as the transform basis) to reduce the number of measurements required for reconstruction [11]. In most cases, the original signal is sparse in a different transform domain, e.g., discrete cosine transform (DCT), discrete wavelet transform (DWT), and so on. Despite being simple and having fast computations, these nonadaptive dictionaries are unable to sparsely represent a given signal of interest well [12]. The approximate solution after the sparse representation of signals still have many nonzero elements. In fact, if an *N*-dimensional signal admits *K*-sparse representation in a dictionary, one can reconstruct exactly the original signal with large probability with *M* = *O*(*Klog*(*N*/*K*)) measurements [10,13]. This shows that only a small number of measurements are necessary to accurately recover the original signal when the signal exhibits a sparser representation. In our previous work [14], we presented an effective sparse representation approach for wireless channels based on the modified Takenaka–Malmquist (TM) basis, under which the represented wireless channel exhibits a sparser structure than the existing sparsifying dictionaries. As the results shown, the approach facilitates to improve the performance of CS. In this paper, we construct a sparse dictionary using the TM basis functions. Compared with other sparse bases, the signal of interest can be represented more accurately with only a limited number of TM basis functions.

The recent growing trend involves replacing the conventional random sensing matrices by optimized ones in order to enhance CS performance further [15,16,17,18,19]. Some conditions have been put forth to evaluate the qualities of a sensing matrix to guarantee exact signal recovery. Candès et al. introduced the restricted isometry property (RIP) and proved that if a sensing matrix satisfies RIP, the original signal can be recovered with high probability [13]. However, it is difficult to verify whether a given sensing matrix satisfies RIP. Another relatively simple property is the mutual coherence [15,16,17,18,19,20]. The coherence of matrix *A* means the maximum absolute correlation between different columns of *A*. Gaussian and Bernoulli matrices are widely used due to their entries being generated by an independent identically distributed (i.i.d.) process and satisfying the RIP. However, it shows low recovery accuracy in the CS system when using the class of matrices as a sensing matrix. In order to improve the CS performance, researchers attempt to optimize the sensing matrix with the hope of increasing the reconstruction probability and taking a fewer number of measurements [15,16,17,18]. Elad proposed an optimized algorithm that iteratively minimizes the t-averaged mutual coherence using shrinkage operation by singular value decomposition (SVD) [15]. However, Elad’s method is time-consuming and creates some large values of coherence. Duarte-Carvajalino and Sapiro proposed a framework for a joint sensing matrix and dictionary design and optimization [16]. The method addressed the problem by making the Gram matrix as close as possible to an identity matrix. Their proposed approach is noniterative and does not have a clear meaning after multiple approximation procedures. In this paper, we proposed a method based on the equiangular tight frame (ETF) design. The optimization objective is to make the Gram matrix of the sensing matrix as close as possible to an ETF. For clarity, the threefold main contributions of this paper are summarized as follows:

- It is revealed that the existing dictionaries are unable to sparsely represent a given signal well. The TM functions have adaptability and robustness in system identification, which invoked us to use it as a basis to build a new dictionary for the sparse representation of a given signal. The simulation results illustrate that compared to other dictionaries, using the constructed dictionary to represent a given signal can obtain a lower mean square error (MSE) under the same number of atoms.
- In order to improve the CS performance further, an ETF-based iterative minimization algorithm is proposed for optimizing the measurement matrix. It is shown that the approach leads to a lower coherence of the equivalent dictionary and significantly improves the signal recovery accuracy than the methods in [15,16].
- The reconstruction quality obtained with our constructed dictionary and the optimal measurement matrix by our approach is superior to that obtained with the existing dictionary and random measurement matrix (such as wavelets or DCT). Moreover, simulation experiments show that after the random measurement matrix optimized by our proposed algorithm, the reconstruction success rate is also improved.

The rest of the paper is organized as follows. In Section 2, we review the basic principles of the CS framework. Section 3 introduces the TM basis function and describes how to construct a dictionary for sparse representation. An ETF-based iterative minimization method is developed in Section 4 for optimizing the measurement matrix. Experimental results are provided in Section 5. Section 6 concludes the paper.

## 2. Background and Preliminaries

CS states that it can enable exact signal reconstruction from far fewer samples than required by the classical Nyquist–Shannon sampling theorem if the signal admits a sparse representation in a certain domain. Consider a signal $x\in {\mathbb{R}}^{N}$, which is sparse over the transformation basis matrix $\Psi \in {\mathbb{R}}^{N\times N}$. Let ***s*** be the sparse representation of x over the basis matrix **Ψ**. Accordingly, **x** can be described as

(1) x=Ψs,

where the signal **x** is said to be *K*-sparse in the **Ψ** domain if ${\left\|s\right\|}_{0}=K\;\left(K\ll N\right)$. The $\ell_{0}$-norm used here denotes the number of the nonzero elements in ***s***. 

The basic model of CS is as follows:

(2)  y=Φx,

where the resulting signal vector $y\in {\mathbb{R}}^{M\times 1}$ is called the measurement vector, $x\in {\mathbb{R}}^{N\times 1}$ is the given signal of interest, and $\Phi \in {\mathbb{R}}^{M\times N}$ (with *M* × *N*) is called the measurement matrix. Since the original signal **x** is sparse in the **Ψ** domain, we can rewrite the measurement model as

(3) y=ΦΨs=As,

where $A=\Phi \Psi \in {\mathbb{R}}^{M\times L}\;\left(L\geq N\geq M\right)$ represents the sensing matrix, $\Psi \in {\mathbb{R}}^{N\times L}$ is the sparsifying basis, e.g., an orthonormal or overcomplete dictionary.

To recover the high-dimensional original signal from the low-dimensional measurement data, it is necessary to solve the following minimization problem

(4) $$\underset{s}{\min}{\left\|s\right\|}_{0}\;subject\;to\;y=As.$$

In general, the reconstruction problem in (4) is a nondeterministic polynomial hard (NP-hard) problem. Fortunately, research in [21] and [22] has demonstrated that $\ell_{1}$-norm minimization is similar to sparse solutions with $\ell_{0}$-norm minimization. Therefore, the $\ell_{1}$-norm is used to substitute for the $\ell_{0}$-norm in (4). The solution to the above problem is the same as the one to the $\ell_{1}$-norm minimization below

(5) $$\underset{s}{\min}{\left\|s\right\|}_{1}\;subject\;to\;y=As$$

where (5) can be solved efficiently using convex relaxation algorithm such as basis pursuit (BP) [23]. Another strategy to address the problem in (4) is to use greedy algorithms, such as the orthogonal matching pursuit (OMP), to find a local optimal solution through multiple iterations [24].

The premise of CS is to ensure that the original signal is sparse or can be represented sparsely. The well-known RIP provides a sufficient condition for an exact or approximate recovery of a sparse representation ***s*** from the low-dimensional measurements ***y***. An M × L sensing matrix ***A*** is said to satisfy the RIP of order *K* if there exists a restricted isometry constant *δ_K_* (0 < *δ_K_* < 1), such that

(6) $$\left(1-\delta_{K}\right){\left\|s\right\|}_{2}^{2}\leq {\left\|s\right\|}_{2}^{2}\leq \left(1+\delta_{K}\right){\left\|s\right\|}_{2}^{2},$$

where ${\left\|s\right\|}_{0}=K$.

RIP measures the degree to which each submatrix consisting of the most *K* columns of ***A*** is close to being an isometry. However, the RIP is too strict and difficult to be used for proving whether a sensing matrix ***A*** satisfies the condition or not. Therefore, the most known constructions of matrices satisfying RIP are random matrices, including the Gaussian matrix, Bernoulli matrix, and almost other matrices with i.i.d. entries. Another relatively simple and feasible property for evaluating the quality of sensing matrices is the mutual coherence. It requires that the rows of measurement matrix **Φ** cannot sparsely represent the columns of the sparse basis **Ψ** and vice versa. The mutual coherence *μ*(***A***) is defined as the largest absolute normalized inner product between different columns in sensing matrix ***A***. Formally, that is expressed as

(7) $$\mu \left(A\right)=\underset{1\leq i,j\leq L,\;i\neq j}{\max}\frac{\left|a_{i}^{T}a_{j}\right|}{\|a_{i}\|{}_{2}\cdot \|a_{j}\|{}_{2}},$$

where $a_{i}$ is the *i*-th column of ***A***.

The mutual coherence can intuitively reflect the quality of a sensing matrix and play important role in the signal reconstruction performance. A smaller value of μ(A) means that the measurement matrix **Φ** and the sparsifying dictionary **Ψ** are less relevant. It means that the two matrices are orthogonal if *μ*(***A***) equals to zero.

An alternative way to measure the mutual coherence is considering the corresponding Gram matrix $G={\tilde{A}}^{T}\tilde{A}$, in which $\tilde{A}$ is obtained by normalizing each column of ***A***. The maximum value of the off-diagonal elements in ***G*** denotes the mutual coherence, that is

(8) $$\mu \left(A\right)=\underset{1\leq i,j\leq L,\;i\neq j}{\max}\left|g_{ij}\right|.$$

Generally, a *K*-sparse signal ***s*** can be recovered from (3) by (4) or (5) if the following inequality holds [25,26,27]:

(9) $$K<\frac{1}{2}\left(1+\frac{1}{\mu \left(A\right)}\right).$$

Even though in many situations a small *μ*(***A***) is desired, the following well-known result of Welch indicates that *μ*(***A***) is bounded below:

(10) $$\mu \left(A\right)\geq \mu_{E}=\sqrt{\frac{L-M}{M\left(L-1\right)}}.$$

It should be noted that the Welch bound *μ_E_* is approximately equal to $\frac{1}{\sqrt{M}}$ when *L* ≥ *M*.

## 3. Construction Procedure of the Dictionary

In this section, we briefly introduce and analyze the Takenaka–Malmquist basis functions. In addition, we show how to construct a new dictionary using the TM basis functions for sparse representation in CS. The preliminary results show the representation advantages of this new approach.

### 3.1. The Takenaka–Malmquist Functions

The TM basis functions were first widely used in the field of control engineering. Extensive research has examined how the TM basis is suitable for linear time-invariant (LTI) system identification [14,28,29,30]. For a deterministic signal, we can learn from the idea of system identification and represent it using the TM basis. The TM functions are defined as follows:

(11) $$F_{k}\left(z\right)=\frac{\sqrt{1-{\left|b_{k}\right|}^{2}}}{z-b_{k}}\overset{k-1}{\underset{i=1}{\prod }}\frac{1-{\bar{b}}_{i}z}{z-b_{i}},\;\;k=1,\;2,\cdots$$

where $b_{i}\in \mathbb{C},\left|b_{i}\right|<1$, ℂ represents an open unit circle in the complex plane, and ${\bar{b}}_{i}$ denotes the conjugation of *b_i_*.

The TM basis functions are constructed to be orthonormal by using the Gram–Schmidt procedure. According to the Cauchy integral formula, it is easy to prove the orthogonality between TM basis functions by

(12) $$\langle F_{k},F_{1}\rangle =F_{k}\left(1/{\bar{b}}_{1}\right)\sqrt{1-{\left|b_{1}\right|}^{2}}=0,\forall k>1$$

which forces *F_k_*(*z*) to have a zero at $1/{\bar{b}}_{1}$.

The orthogonality between the TM basis functions allowed it to serve as a dictionary, from which the representation coefficients of a signal could be efficiently selected. Hence, only a limited number of linear combinations of TM basis functions are required to approximate the original signal. This provides us with a theoretical basis and feasibility to build a dictionary next.

### 3.2. Construction of the Dictionary

The number of nonzero terms *K* of the sparse representation coefficient ***s*** of a signal **x** in a sparsifying dictionary **Ψ** is a very important factor. The smaller the value of *K*, the fewer the number of measurements are required for signal recovery. Therefore, the sparsifying dictionary **Ψ** should be properly selected so that the signal has a sparser representation structure. In other words, *K* should be reduced as close to zero as possible. The most commonly used approach is using basis function to construct a sparsifying dictionary, such as DWT, DCT, and so forth. For a given dictionary, a signal is approximately represented as a linear combination of the dictionary atoms:

(13) $$x\approx \sum_{k=1}^{n}s_{k}\psi_{k}.$$

The approximate solution after the sparse representation of signals in a DCT (or DWT) domain still have many nonzero elements. Under the same number of atoms, the approximate representation of the signal has a large error. To further improve the sparse representation performance, we construct a sparsity dictionary by using the TM basis functions. The detailed construction process is as follows.

For a given signal $x\in {\mathbb{R}}^{N}$, *N* poles are uniformly selected in the unit circle, and then the poles are set as the poles of the TM basis functions, forming an initial sparsifying dictionary $\Psi =\left[F_{1}\left(z\right),\cdots ,F_{\xi }\left(z\right),\cdots ,F_{N}\left(z\right)\right]$. Then, the TM basis function corresponding to each pole takes $\omega_{k}=\frac{2\pi \left(k-1\right)}{N},k=1,2,\cdots ,N$ as the sampling frequency to sample the signal **x** at equal intervals. In this way, the column vector corresponding to each TM basis function is set by

(14) $$F_{\xi }=\left[\begin{array}{c} F_{\xi }\left(e^{j0}\right)\\ \vdots \\ \begin{array}{c} F_{\xi }\left(e^{j\omega_{k}}\right)\\ \vdots \\ F_{\xi }\left(e^{j\frac{2\pi \left(N-1\right)}{N}}\right) \end{array} \end{array}\right].$$

Therefore, the constructed dictionary can be expressed as follows

(15) $$\Psi =[\begin{array}{ccccc} F_{1}\left(e^{j0}\right) & \cdots & F_{\xi }\left(e^{j0}\right) & \cdots & F_{N}\left(e^{j0}\right)\\ \vdots & \ddots & \vdots & \ddots & \vdots \\ F_{1}\left(e^{j\omega_{k}}\right) & \cdots & F_{\xi }\left(e^{j\omega_{k}}\right) & \cdots & F_{N}\left(e^{j\omega_{k}}\right)\\ \vdots & \ddots & \vdots & \ddots & \vdots \\ F_{\mathrm{1;}}\left(e^{j\frac{2\pi \left(N-1\right)}{N}}\right) & \cdots & F_{\xi }\left(e^{j\frac{2\pi \left(N-1\right)}{N}}\right) & \cdots & F_{N}\left(e^{j\frac{2\pi \left(N-1\right)}{N}}\right) \end{array}].$$

**Figure 1:**
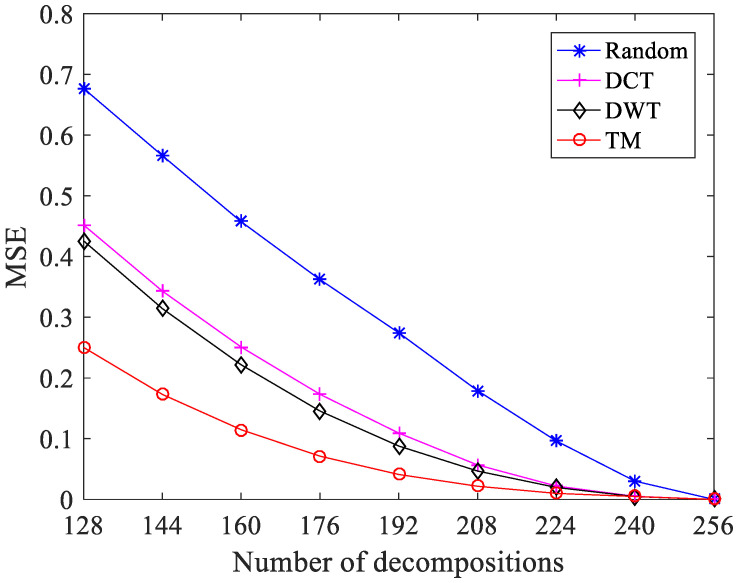
Comparison between the four sparsifying dictionaries under the different number of decompositions using orthogonal matching pursuit (OMP).

To illustrate the performance of the constructed dictionary, we provide a comparison of the results in Figure 1.

As a performance measurement between the original signal and the reconstructed signal, the MSE is defined as follows:

(16) $$MSE=\frac{1}{N}\sum_{K=1}^{N}{\left\|x-\bar{x}\right\|}_{2}^{2}.$$

Without loss of generality, a Rayleigh-distributed signal is taken here as an example. The length of the signal is 256. A random dictionary of size 256 × 256 is considered, which is drawn from i.i.d. zero mean and unit variance Gaussian distribution. The DCT, DWT, and the constructed TM dictionary of the same dimension are compared. The OMP algorithm is used to compare the MSE of the reconstructed signal after the sparse representation of the original signal under different numbers of decomposed atoms. The results demonstrate that compared with the random, DCT, and DWT dictionaries, using the constructed TM dictionary to represent a given signal can get lower MSE under the same number of decompositions.

## 4. Optimizing the Measurement Matrix

In this section, we investigate the problem of optimizing the measurement matrix given a sparsifying dictionary to increase CS performance further. To address the problem, an ETF-based iterative minimization algorithm is proposed.

The object is to optimize the measurement matrix **Φ** to minimize the mutual coherence *μ*(***A***). In other words, it is to find a measurement matrix **Φ** making the corresponding Gram matrix as close to an identity matrix as possible. The column-normalized form of the sensing matrix ***A*** is called an ETF. However, it is often that such an ETF does not exist for any arbitrary dimension [25,31]. Therefore, our optimization goal is to make the sensing matrix ***A*** as close as possible to an ETF. The minimization problem is formulated as follows:

(17) $$\underset{\Phi ,H\epsilon \Lambda_{set}}{\min}{\left\|A^{T}A-H\right\|}_{F}^{2}.$$

where ${\left\|\cdot \right\|}_{F}$ is the Frobenius norm. The $\Lambda_{set}$ denotes a convex set that leads the optimization problem to have solutions. For any arbitrary dimension, $\Lambda_{set}$ is regularized as

(18) $$\Lambda_{set}=\left\{H\in {\mathbb{R}}^{L\times L}:H=H^{T},diag\;H=1,\underset{i\neq j}{\max}\left|h_{ij}\right|\leq \mu_{E}\right\}.$$

As mentioned before, *μ_E_* is the Welch bound and is used here as a prescribed parameter.

The alternate optimization method is usually used to address the minimization problem in (17). The difference between our proposed method and others is that we assign weight values to the Gram matrix of the previous iteration as well as the Gram matrix of the current iteration in the stage of updating the Gram matrix. We modify the singular value without changing the properties of the Gram matrix, so that the minimum singular value of the updated Gram matrix increases and the maximum singular value decreases, and then the linear independence between the column vectors of the Gram matrix is enhanced. In this way, a new diagonal matrix is obtained by using the eigenvalue averaging method for the updated Gram matrix to construct an optimized measurement matrix. The detailed optimization procedure is shown in Algorithm 1.

In this algorithm, the Gram matrix of the normalized sensing matrix is computed. In order to make it close to an ETF, the Gram matrix is projected onto the convex set Λ*set* by the shrinking operation function:

(19) $$g_{ij}=\{\begin{array}{ll} 1 & i=j\\ g_{ij} & i\neq j,\left|g_{ij}\right|\leq \mu_{E}\\ sign\left(g_{ij}\right)\cdot \mu_{E} & i\neq j,\left|g_{ij}\right|\mu_{E} \end{array}.$$

Since the Gram matrix is projected onto Λ*set*, its structure changes significantly. In order to retain some of its feature information and approximate the ETF, we give weights to the Gram matrix before and after the update:

(20) $$G_{\left(t+1\right)}=\beta G_{\left(t\right)}+\left(1-\beta \right)G_{\left(t-1\right)}.$$

In general, the aforementioned shrinking operation causes the rank of the Gram matrix to be full. Therefore, the next step is to force a rank *M* by a singular value decomposition of the obtained Gram matrix, i.e., **UΛV***^T^ =* **G**, in which rank(Λ)=M≪L. Let $\lambda_{1},\lambda_{2},\cdots ,\lambda_{M}$ be the eigenvalues of the diagonal matrix Λ. According to singular value decomposition, the smaller the maximum singular value, the better the incoherence of the matrix. Therefore, in order to reduce the mutual coherence between the different columns of ***A***, we modify the eigenvalues by averaging all eigenvalues of the obtained Gram matrix and get a new diagonal matrix $\tilde{\Lambda }$. Let ${\tilde{\lambda }}_{i}\left(i=1,2,\cdots ,M\right)$ be the diagonal elements of $\tilde{\Lambda }$, and ${\tilde{\lambda }}_{i}=\frac{1}{M}\overset{M}{\underset{i=1}{\sum }}\lambda_{i}$. Then, we build the squared root of the obtained Gram matrix, **G** = **S***^T^***S**, where $S=\sqrt{\tilde{\Lambda }}V^{T}$. Now, the solution of optimizing the measurement matrix is equivalent to solving

(21) $$\underset{\Phi }{\min}{\left\|S-\Phi \Psi \right\|}_{F}^{2}.$$

The solution process is as follows:

(22) $$\begin{array}{ll} {\left\|S-\Phi \Psi \right\|}_{F}^{2} & =tr\left[{\left(S-\Phi \Psi \right)}^{T}\left(S-\Phi \Psi \right)\right]\\ & =tr\left(S^{T}S\right)-2tr\left(\Psi^{T}\Phi^{T}S\right)+tr\left(\Psi^{T}\Phi^{T}\Phi \Psi \right). \end{array}$$

Here, tr(⋅) denotes the matrix trace operation.

(23) $$\begin{array}{ll} \frac{\partial {\left\|S-\Phi \Psi \right\|}_{F}^{2}}{\partial \Phi } & =-2\frac{\partial tr\left(\Psi^{T}\Phi^{T}S\right)}{\partial \Phi }+\frac{\partial tr\left(\Psi^{T}\Phi^{T}\Phi \Psi \right)}{\partial \Phi }\\ & =-2\frac{\partial tr\left(\Phi^{T}S\Psi^{T}\right)}{\partial \Phi }+\frac{\partial tr\left(\Phi \Psi \Psi^{T}\Phi^{T}\right)}{\partial \Phi }\\ & =-2S\Psi^{T}+2\Phi \Psi \Psi^{T}. \end{array}$$

Solved by least squares, it is now easy to find the optimized solutions $\Phi =\sqrt{\tilde{\Lambda }}V^{T}\Psi^{†}$, where † denotes pseudoinverse.

**Algorithm 1:** The proposed optimization algorithm.**Input:** The given sparsifying dictionary $\Psi \in {\mathbb{R}}^{N\times L}$,the number of iterations ***Iter***, and threshold *μ*_*E*_.
**Output:** The measurement matrix **Φ**.
1: **Initialization:** Set $\Phi \in {\mathbb{R}}^{M\times N}$ to be a random matrix.
2: **for**
*l* = 1 : ***Iter* do**
3:  Normalize the columns of the sensing matrix ***A*** = **ΦΨ** to obtain ***Ã***.
4:  Compute the Gram matrix ***G*** = ***Ã_*T*_Ã***.
5:  Update the Gram matrix by the shrinking operation (19).
6:  Assign weight values between the previous and current Gram matrix to update the Gram matrix: **G**_(*t* + 1)_ = *β***G**_(*t*)_ + (1 − *β*)**G**_(*t* − 1)_.
7:  Apply SVD **UΛV**^*T*^ = **G** and force the rank of **G** to be equal to *M*.
8:  Average the eigenvalues to get new diagonal matrix $\tilde{\Lambda }$.
9:  Update the measurement matrix $\Phi =\sqrt{\tilde{\Lambda }}V^{T}\Psi^{†}$.
10: **end for**


## 5. Experimental Results

In this section, we carry out various numerical simulations to support the effectiveness of the proposed approach. The experiments include a performance evaluation of the proposed algorithm and a performance comparison of different sparsifying dictionaries. In Section 5.1, we present some experiments to illustrate the performance of the proposed algorithm and compare it with other algorithms. In Section 5.2 we demonstrate the superiority of the CS system designed with the constructed sparsifying dictionary over the existing ones under the same measurement matrix. In addition, the signal recovery quality are evaluated to compare the performance of the different sparsifying dictionaries under the optimized measurement matrix using our proposed approach.

### 5.1. Performance Evaluation of the Proposed Algorithm

In this subsection, the performance of the proposed algorithm in the CS system is evaluated. Several comparison simulations are given to verify the effectiveness of the proposed algorithm in terms of the measures *μ*(***A***) and the signal recovery performance. Here, a random dictionary (every entry is drawn from i.i.d. zero mean and unit variance Gaussian distribution) of size 256 × 400 was used. We generated a Gaussian matrix with a size of 64 × 256 as a random measurement matrix and used it as an initial condition to obtain the corresponding optimized measurement matrix by using the proposed algorithm and the algorithm in [15,16], respectively.

The parameter settings in the abovementioned algorithms are shown as follows. For Elad’s algorithm, the parameters *t*, *γ* are set to 0.2 and 0.55, respectively. The iteration number *Iter* is set to 30 for both Elad’s and our algorithm. It is worth noting that the Duarte-Carvajalino and Sapiro algorithm is noniterative.

**Figure 2:**
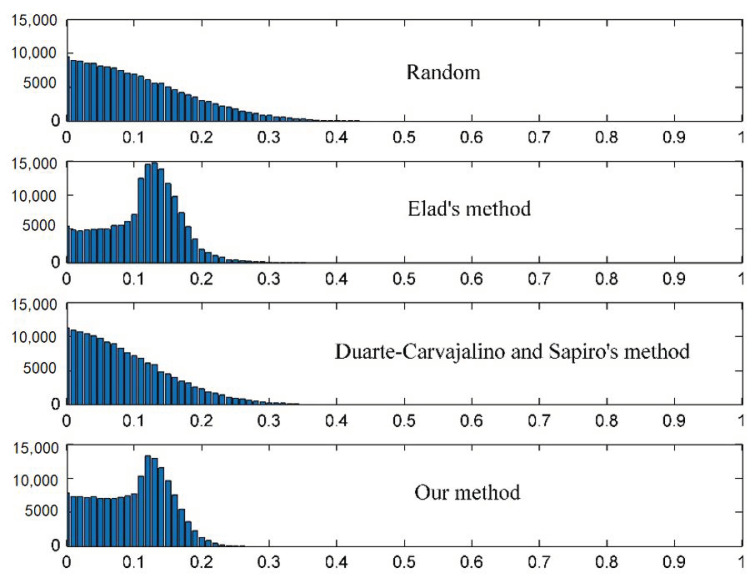
Histogram of the absolute off-diagonal entries of the corresponding normalized Gram matrix to each of the four sensing matrices.

Since the corresponding Gram matrices with smaller nonzero terms result in good reconstruction performance, the local cumulative coherence values can be used to evaluate the performance of the sensing matrix. The histogram of the absolute off-diagonal entries of the corresponding normalized Gram matrix to each sensing matrix is depicted in Figure 2. As can be seen, both the method proposed in [15,16] and our proposed method reduce the number of large absolute values in the Gram matrix. Compared with Elad’s method and Sapiro’s method, it easy to find in Figure 2 that the largest absolute values are smaller. Furthermore, the histogram of our method shows that the local cumulative mutual coherence values shift significantly to the left and concentrate within 0.2, which means that the local coherence is also smaller.

**Figure 3:**
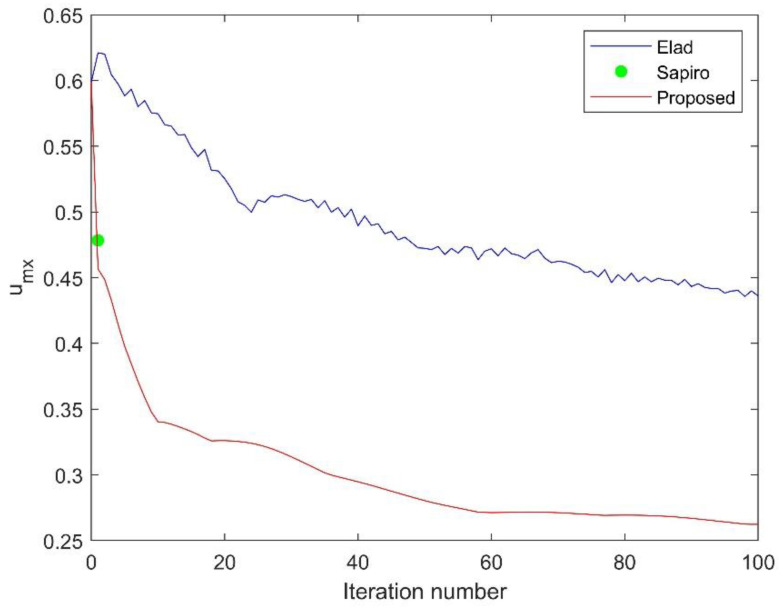
Evolution of the mutual coherence versus iteration number.

To illustrate the behavior of the proposed algorithm, the evolution of the mutual coherence versus iteration number is shown in Figure 3. Since Sapiro’s method is noniterative, its optimized value is shown as a point in the graph. Both Elad’s method and our proposed method demonstrate the mutual coherence value decreases with the number of iterations. However, our method shows that the rate of decline is significantly faster. Moreover, the final value of *μ*(***A***) is also smaller than the first two.

Next, the signal reconstruction performance via the abovementioned measurement matrices are further investigated. We use the percentage of successful recovery to evaluate the sparse signal recovery quality. It is considered to be a successful recovery if the reconstruction error ${\left\|x-\hat{x}\right\|}_{2}/{\left\|x\right\|}_{2}<10^{-3}$. The known OMP algorithm is used for signal recovery.

**Figure 4:**
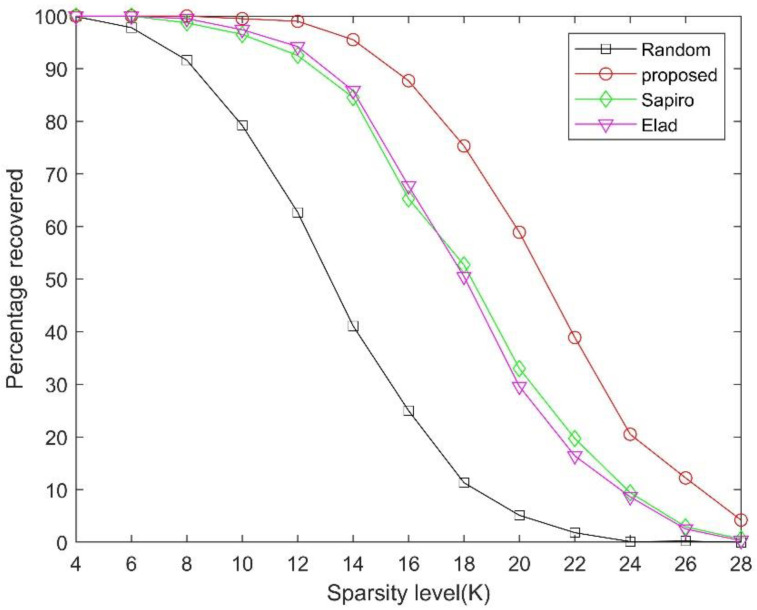
Comparison of the reconstruction performances using various measurement matrices under the same measurements *M* = 64.

In the first experiment, we generate 1000 *K*-sparse vectors with nonzero entries chosen at random for each sparsity level *K*. These sparse vectors are used as test signals to evaluate the CS performance. Figure 4 shows the signal reconstruction performance of the CS system before and after the measurement matrix optimization using different optimization methods, with signal sparsity level *K* varying from 4 to 28 and *M =* 64. As can be seen, all the optimization methods lead to improved CS performance. In addition, the result shown in Figure 4 indicates that the proposed method performs better than both Elad’s method and Duarte-Carvajalino and Sapiro’s method.

**Figure 5:**
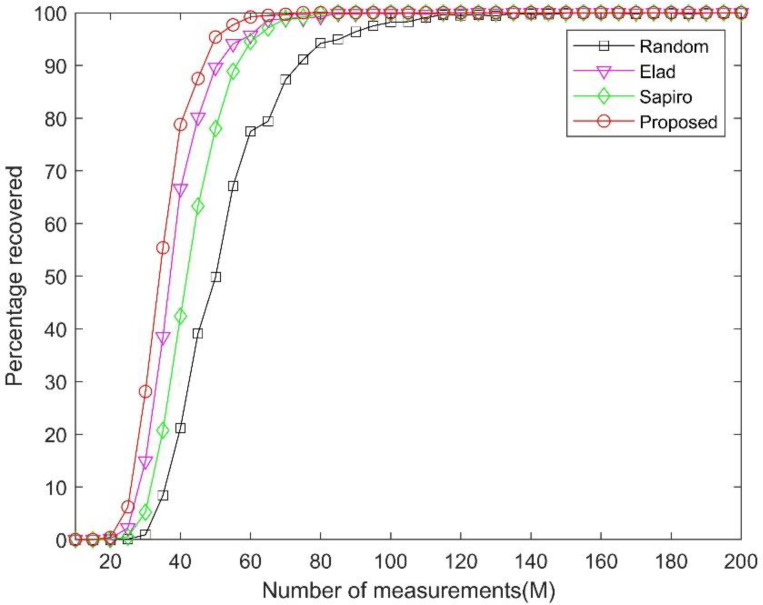
Comparison of the reconstruction performances using various measurement matrices under the same sparsity level *K* = 10.

In the second experiment, the sparsity level *K* is fixed, and the measurement value *M* is changeable. Figure 5 shows the comparison of the reconstruction performances using various measurement matrices under the same sparsity level *K* = 10. As the number of measurements increases, the percentage of signal reconstruction also increases. As expected, it is shown in Figure 5 once again that the proposed method outperforms the other two methods.

### 5.2. Performance Comparison of Different Sparsifying Dictionaries

In this subsection, we investigate the performance of different sparsifying dictionaries in the CS system. As in the previous section, we quantify the performance by measuring the percentage of successful recovery signals. We previously verified that compared to DCT, DWT, and random dictionaries, using the constructed TM dictionary to represent a given signal can get lower MSE under the same number of atoms. Here, we further evaluate the reconstruction performance in CS systems using the constructed TM, DCT, DWT, and random matrices as sparsifying dictionaries.

**Figure 6:**
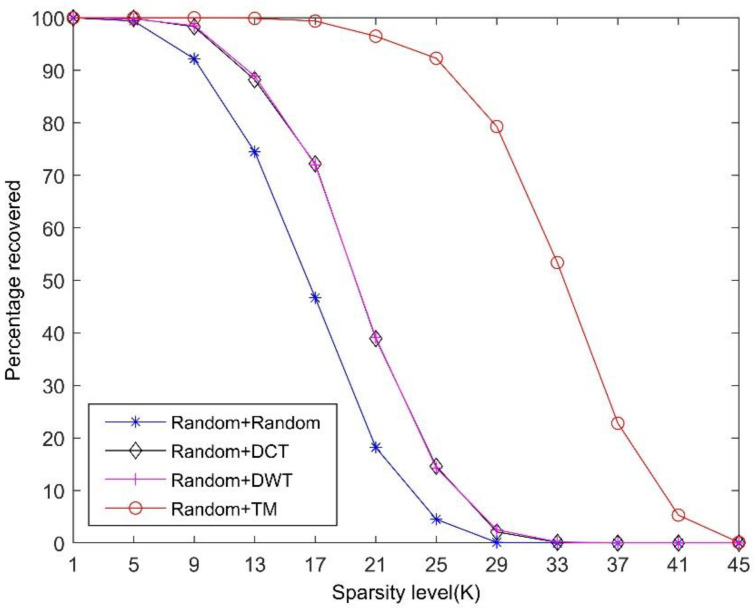
Comparison of the reconstruction performances using different sparsifying dictionaries under the same random measurement matrix (*M* = 64).

We set up a new experiment, in which the dimension of the sparsifying dictionaries is set to 256 × 256. In addition, we generate a measurement matrix $\Phi \in {\mathbb{R}}^{64\times 256}$ with random Gaussian distribution. By multiplying it with the above sparsifying dictionaries, we obtain four sensing matrices. Then, the OMP algorithm is used to recover the signal. In the experiments, we tested 1000 sparse signals in each trial. We evaluated the performance of a sparsifying dictionary in the CS system by the percentage of successful signal recovery. The error threshold between the original signal and the reconstructed signal is the same as in the previous subsection, which is 10^−3^. Figure 6 shows the comparison of the reconstruction performances using different sparsifying dictionaries under the same random measurement matrix. The results demonstrate that the constructed TM dictionary has a better performance in terms of reconstruction quality compared with the random, DCT, and DWT dictionaries. Using TM as a sparsifying dictionary can achieve a higher reconstruction rate among these dictionaries under the same sparsity level.

**Figure 7:**
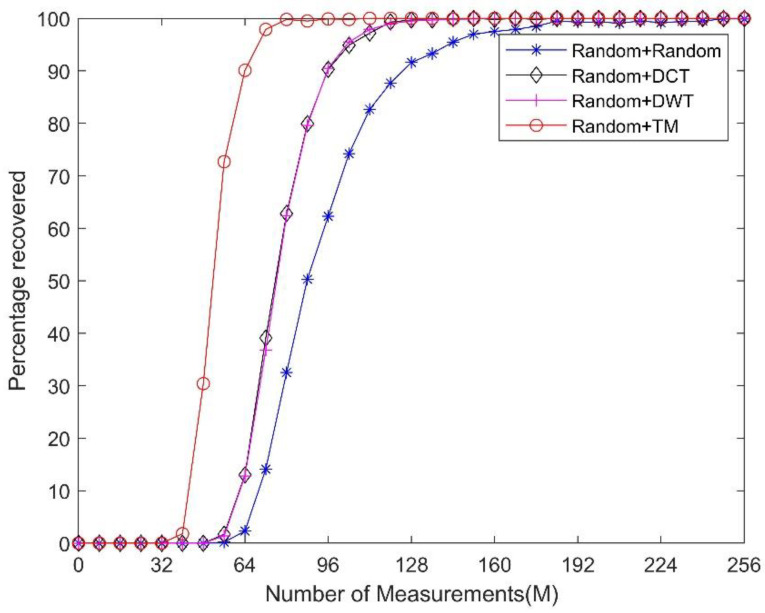
Comparison of the reconstruction performances using different sparsifying dictionaries under the same sparsity level *K* = 25 and the same random measurement matrix.

In addition, we fixed the sparsity level of the signal and performed comparison experiments by changing the measurement numbers of the measurement matrix. The sparsity level *K* is set to 25. Figure 7 reveals that by using the TM dictionary as **Ψ**, one can use a fewer number of measurements to achieve the same performance as that obtained using the DCT, DWT, and random dictionaries.

**Figure 8:**
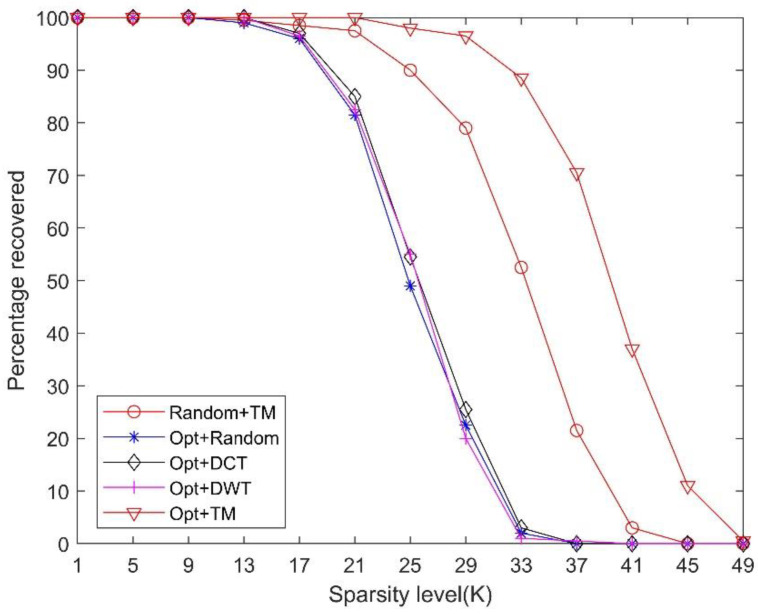
Comparison of the reconstruction performances using different sparsifying dictionaries with the optimized measurement matrix (*M* = 64).

**Figure 9:**
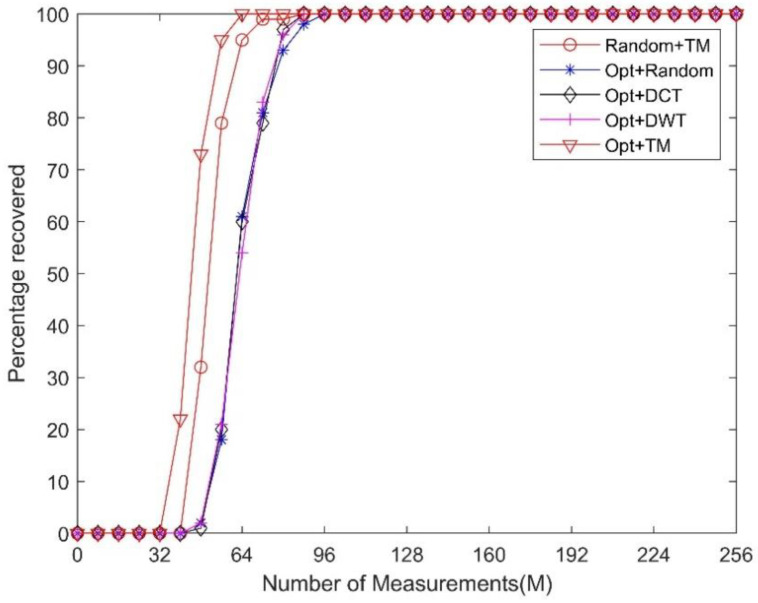
Comparison of the reconstruction performances using different sparsifying dictionaries under the same sparsity level *K* = 25 and the optimized measurement matrix.

To further evaluate the performance of the constructed TM sparsifying dictionary, we used the proposed algorithm to optimize the measurement matrix in order to reduce the mutual coherence between the dictionary and the measurement matrix. Different from previous experiments, we compared the performance of different sparsifying dictionaries in CS systems under an optimized measurement matrix. As shown in Figure 8 and Figure 9, after using the proposed algorithm to optimize the measurement matrix, the performance of the signal recovery is improved. It was observed that using the initial random measurement matrix and the constructed TM dictionary in the CS system can allow us to still obtain a better performance than using the optimized measurement matrices with the random, DCT, and DWT dictionaries. This proves that our constructed TM dictionary is more robust and effective.

## 6. Conclusions

In this paper, we constructed a novel sparsifying dictionary using the TM functions for the sparse representation of a given signal. The advantages are higher performance in sparsely representing signals of interest at the same degree of atomic decomposition. Our proposed iterative algorithm is based on the equiangular tight frame, for which its related mutual coherence between the measurement matrix and the sparsifying dictionary is reduced. Simulation results demonstrated the promising performance of the algorithm and the superiority of the CS system, designed with the constructed dictionary and the optimized measurement matrix, over the existing ones in terms of signal recovery accuracy.

In the future, we will try to combine the constructed sparsifying dictionary with the measurement matrix to optimize both simultaneously. We will also try to apply our method in practical applications, such as channel estimation. Further investigation of this topic is needed.
